# Identifying ceRNA Networks Associated With the Susceptibility and Persistence of Atrial Fibrillation Through Weighted Gene Co-Expression Network Analysis

**DOI:** 10.3389/fgene.2021.653474

**Published:** 2021-06-23

**Authors:** Yaozhong Liu, Na Liu, Fan Bai, Qiming Liu

**Affiliations:** Department of Cardiovascular Medicine, Second Xiangya Hospital, Central South University, Changsha, China

**Keywords:** atrial fibrillation, susceptibility, persistence, ceRNA, WGCNA, RWR-M

## Abstract

**Background:** Atrial fibrillation (AF) is the most common arrhythmia. We aimed to construct competing endogenous RNA (ceRNA) networks associated with the susceptibility and persistence of AF by applying the weighted gene co-expression network analysis (WGCNA) and prioritize key genes using the random walk with restart on multiplex networks (RWR-M) algorithm.

**Methods:** RNA sequencing results from 235 left atrial appendage samples were downloaded from the GEO database. The top 5,000 lncRNAs/mRNAs with the highest variance were used to construct a gene co-expression network using the WGCNA method. AF susceptibility- or persistence-associated modules were identified by correlating the module eigengene with the atrial rhythm phenotype. Using a module-specific manner, ceRNA pairs of lncRNA–mRNA were predicted. The RWR-M algorithm was applied to calculate the proximity between lncRNAs and known AF protein-coding genes. Random forest classifiers, based on the expression value of key lncRNA-associated ceRNA pairs, were constructed and validated against an independent data set.

**Results:** From the 21 identified modules, magenta and tan modules were associated with AF susceptibility, whereas turquoise and yellow modules were associated with AF persistence. ceRNA networks in magenta and tan modules were primarily involved in the inflammatory process, whereas ceRNA networks in turquoise and yellow modules were primarily associated with electrical remodeling. A total of 106 previously identified AF-associated protein-coding genes were found in the ceRNA networks, including 16 that were previously implicated in the genome-wide association study. Myocardial infarction–associated transcript (MIAT) and LINC00964 were prioritized as key lncRNAs through RWR-M. The classifiers based on their associated ceRNA pairs were able to distinguish AF from sinus rhythm with respective AUC values of 0.810 and 0.940 in the training set and 0.870 and 0.922 in the independent test set. The AF-related single-nucleotide polymorphism rs35006907 was found in the intronic region of LINC00964 and negatively regulated the LINC00964 expression.

**Conclusion:** Our study constructed AF susceptibility- and persistence-associated ceRNA networks, linked genetics with epigenetics, identified MIAT and LINC00964 as key lncRNAs, and constructed random forest classifiers based on their associated ceRNA pairs. These results will help us to better understand the mechanisms underlying AF from the ceRNA perspective and provide candidate therapeutic and diagnostic tools.

## Introduction

Atrial fibrillation (AF) is the most common type of cardiac arrhythmia and poses a significant burden to patients and physicians (Hindricks et al., 2020). The currently estimated global prevalence of AF is between 2 and 4% (Hindricks et al., 2020; Virani et al., 2020), and by the middle of the 21st century, AF will be diagnosed in an estimated 72 million individuals in Asia, 16 million in America, and 14 million in Europe (Kornej et al., 2020). Well-known risk factors contribute to AF susceptibility, including aging, male sex, alcohol consumption, obesity, and smoking as well as comorbidities such as heart failure, diabetes, obstructive sleep apnea, and inflammatory disease (Chung et al., 2020). AF is also heritable (Weng et al., 2017), and two large-scale genome-wide association studies (GWAS) identified more than 100 loci associated with AF (Nielsen et al., 2018; Roselli et al., 2018). AF increases the risk of stroke, dementia, and depression and contributes to a 1.5–3.5-fold increase in mortality (Hindricks et al., 2020). Despite its epidemiological importance, the fundamental mechanisms that underlie AF remain poorly understood.

The basic mechanisms that underlie AF include ectopic firing and reentry circuits, both of which are associated with atrial electrical and structural remodeling (Iwasaki et al., 2011). Electrical remodeling refers to changes in the expression or function of the ion channels that affect the electrical activity of cardiomyocytes, whereas structural remodeling refers to alterations that occur in the tissue architecture, such as atrial fibrosis and dilation (Nattel and Harada, 2014). Both electrical and structural remodeling provide the substrates for ectopic firing and reentry circuits. Some newly proposed physiological processes, including oxidative stress, inflammation, and mitochondrial damage, have the potential to trigger atrial remodeling and represent promising therapeutic targets in AF (Nattel et al., 2020).

Depending on the presentation, duration, and spontaneous termination of AF, five patterns have been classified, including first-diagnosed, paroxysmal, persistent, long-standing persistent, and permanent AF (Hindricks et al., 2020). The self-perpetuating nature of AF represents a major challenge that has limited the success of pharmacological or ablation therapies and might serve as the leading mechanism that contributes to the development of paroxysmal to persistent to permanent AF (Nattel et al., 2014). Rapid, irregular pacing causes abnormalities to develop in the underlying electrical or structural properties of the atria, and these remodeling events can further promote AF development (Nattel et al., 2014), leading to a vicious cycle of “AF begetting AF.” Therefore, identifying the regulators that mediate the pathogenic biological processes that underlie atrial remodeling has become a primary goal in AF-related clinical and experimental studies. The recent discovery of a new group of mediators, known as competing endogenous RNAs (ceRNAs), offers a unique opportunity for deciphering this complex heart rhythm disorder.

Numerous microRNA (miRNA) binding sites have been identified on messenger RNAs (mRNAs), long non-coding RNAs (lncRNAs), circular RNAs, and pseudogenes (Tay et al., 2014), leading to the hypothesis that RNA transcripts containing miRNA-binding sites can “communicate” or regulate each other by competing for shared miRNAs, acting as ceRNAs (Salmena et al., 2011). This hypothesis has been widely adopted in investigations of the roles played by non-coding RNAs in disease pathogenesis, especially lncRNAs. A great example is cardiac apoptosis–related lncRNA (CARL), which competitively binds to miR-539, preventing the miR-539-dependent downregulation of prohibitin 2 (PHB2), allowing PHB2 to inhibit mitochondrial fission and apoptosis in cardiomyocytes (Wang et al., 2014). However, the functions of ceRNA pairs in AF have not yet been well-illustrated. As the “language” of ceRNAs, many miRNAs have been demonstrated to promote AF development by causing atrial electrical and structural remodeling (Luo et al., 2015), suggesting the existence of ceRNA crosstalk in AF pathogenesis. Previous research has attempted to identify ceRNA pairs associated with AF by identifying differentially expressed lncRNAs/mRNAs between patients with AF and those with sinus rhythm (SR) (Qian et al., 2019). However, differentially expressed gene (DEG) analysis ignores the interconnections between ceRNAs and may filter out genes with high centralities that engage in high levels of ceRNA crosstalk. In addition, DEGs were identified by comparing the expression patterns between a persistent AF and an SR group; however, these genes could be associated with either increased susceptibility or persistence, and the distinction between susceptibility- and persistence-associated ceRNA pairs is not possible in comparisons of AF and SR genes.

The characteristics and underlying molecular mechanisms associated with AF susceptibility might differ from those associated with AF persistence. A previous microarray study (Deshmukh et al., 2015) compared the transcriptomic profiles of left atrial appendages from three types of patients (no AF history, AF history in SR at surgery, AF history in AF at surgery) and found that AF susceptibility was associated with changes in the activities of several transcription factor targets related to inflammation, oxidation, and cellular stress responses, whereas AF persistence was associated with the remodeling of ion channel expression. Many of these changes corroborate the findings of previous clinical and electrophysiology studies of AF. On the one hand, inflammatory disease, cardiovascular comorbidities, and increased serum inflammatory biomarkers have been associated with an increased risk of AF (Chung et al., 2020; Li and Brundel, 2020), which supports the idea that inflammation and oxidative stress contribute to AF susceptibility. On the other hand, GWAS studies and gain and loss of function studies have identified various genes related to ion channel expression that are associated with AF persistence (Roselli et al., 2020). Drugs that target ion channel currents represent the current pharmacological strategies used to treat AF patients. However, the application of anti-inflammatory agents for AF prevention has generally failed to establish AF-specific indications, and the efficiency of anti-arrhythmia drugs in AF treatment remains relatively unsatisfactory (Chung et al., 2020). Therefore, the identification of new regulators to prevent the initiation and progression of this complex arrhythmia could provide new therapeutic and diagnostic targets.

**Figure 1:**
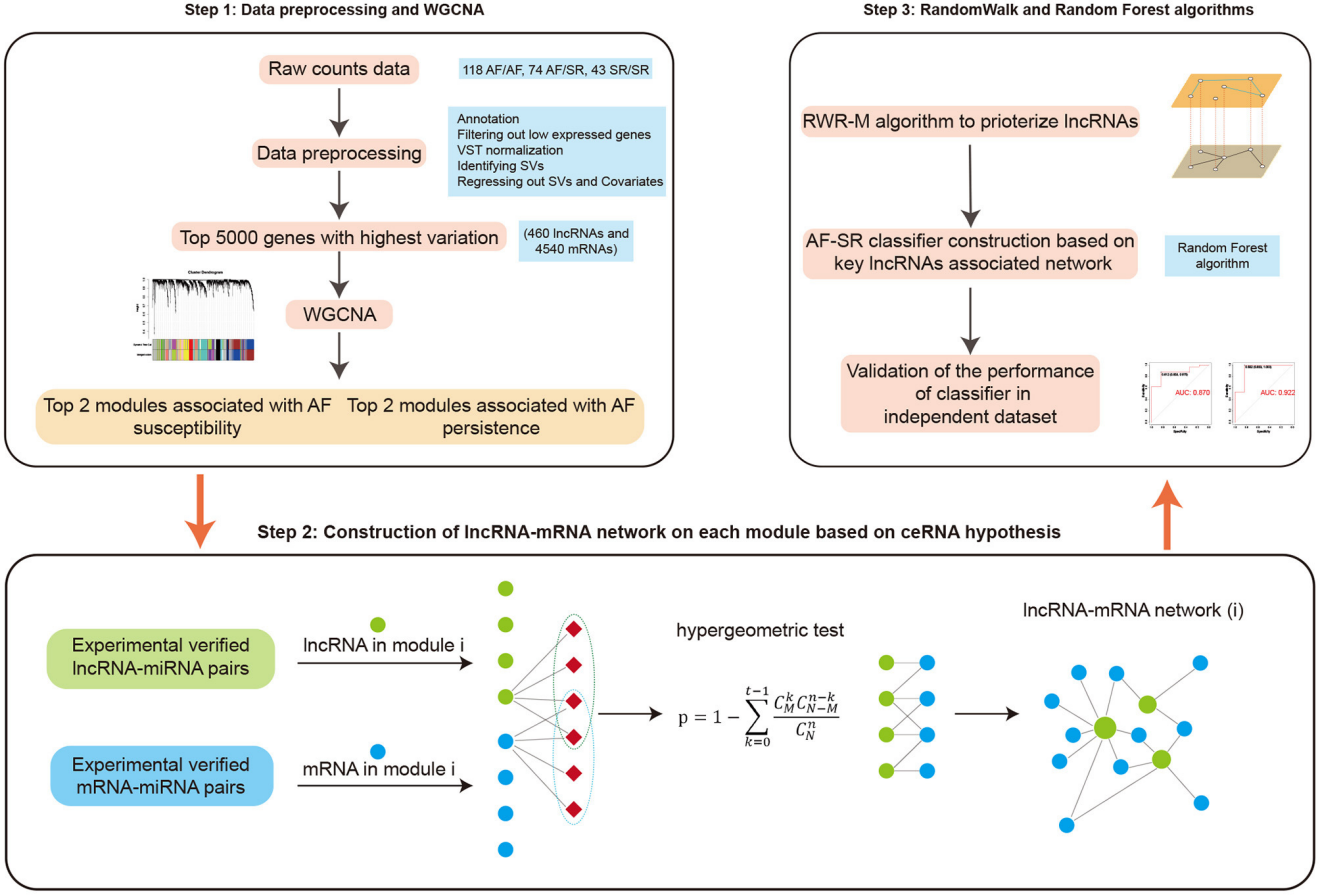
Study workflow.

In this study, we introduce a method known as the weighted gene co-expression network analysis (WGCNA), which can cluster highly correlated genes into association modules and then relate each module to external clinical traits (Langfelder and Horvath, 2008). The construction of a ceRNA network within a disease-related module ensures that the identified nodes are highly co-expressed and enhances the reliability and significance of the network. Our present study used a large cohort (*n* = 235) of left atrial tissue samples derived from patients from three types of atrial rhythm. By analyzing the relationships between the modules and the various phenotypes, we were able to identify AF susceptibility- and persistence-associated ceRNA networks, which will inform future research. We also applied a newly proposed algorithm, known as the random walk with restart on multiple networks (RWR-M), to prioritize lncRNAs by analyzing the “proximity score” of each lncRNA to known disease-related genes. Moreover, by applying the random forest classification algorithm, we demonstrate that key lncRNA-associated ceRNA pairs could distinguish AF from SR patients in both training and independent test sets. In general, our study generates a state-of-the-art pipeline for the construction of disease-associated ceRNA networks and the prioritization of newly identified disease-associated genes (Figure 1). These results provide information to promote a better understanding of the mechanisms that underlie AF from the ceRNA perspective and identify new candidate therapeutic and diagnostic targets.

## Materials and Methods

### Data Acquisition and Preprocessing

The GSE69890 data set was used to obtain the RNA sequencing results generated from left atrial appendage (LAA) tissue samples derived from 235 subjects of European descent (Hsu et al., 2018). These 235 subjects were divided into three groups according to their atrial rhythm phenotypes: no history of AF and in SR at the time of surgery (SR/SR, *n* = 43), a history of AF but in SR at the time of surgery (AF/SR, *n* = 74), and a history of AF and flutter at the time of surgery (AF/AF, *n* = 118). The raw count file was downloaded from the NCBI Gene Expression Omnibus (GEO) database (https://www.ncbi.nlm.nih.gov/). We annotated the Ensemble ID identifier with the Ensembl Release 99 (https://www.ensembl.org/index.html), retaining rows that (1) had an official symbol name and (2) belonged to the lncRNA or mRNA category and removing rows that had duplicated gene names. Genes with counts of <10 in more than 80% of samples were filtered out, and the remaining data were variance-stabilized and transformed using the R package DESeq2 (Love et al., 2014). Surrogate variables (SVs) were calculated using the sva package (Leek et al., 2012), and the expression of each gene was adjusted by the sex covariate and the identified SVs. Finally, the top 5,000 genes with the highest variance were selected for the construction of a gene co-expression network.

For validation, the GSE41177 data set (Yeh et al., 2013), which includes microarray data from a total of 38 left atrial tissues from 6 persistent AF and 32 SR patients, was downloaded from the GEO database. The raw CEL files were obtained and preprocessed using a robust multiarray average (RMA) algorithm with the affy package (Gautier et al., 2004) for background correction and quantile normalization. The median expression values among all multiple probe IDs were selected to represent the corresponding gene.

### Construction of a Weighted Gene Co-Expression Network

WGCNA is one of the most widely used methods for the construction of a gene co-expression network (Langfelder and Horvath, 2008). In this study, we focused only on positive gene correlations; therefore, a signed network was constructed. First, a signed adjacency matrix was generated between genes based on their correlation. The adjacency value, *signed a*_*ij*_, for genes *i* and *j*, is defined as

$$\begin{array}{l} \text{signed }{\text{a}}_{\text{ij}}={\left|\left(1+\text{cor}\left(x_{i},x_{j}\right)\;\right)/2\right|}^{\beta }, \end{array}$$

where *cor*(*x*_*i*_, *x*_*j*_) is the Pearson's correlation coefficient between gene *i* and gene *j*, and β is an integer to let the network display a scale-free topology property. A value of β = 12 was selected in this study because this satisfied a degree of independence of 0.9 with the minimum value. Then, a topological overlap measure (TOM) was created to reduce the network sensitivity to spurious connections or random noise (Ravasz et al., 2002). Hierarchical clustering was performed on the matrix 1 – TOM and the dynamic tree cut method was applied to generate modules of highly co-expressed genes with parameters set to a deepSplit of 2, minModuleSize of 30, and height cutoff of 0.25 as recommended (Zhang and Horvath, 2005). The module eigengene (ME) is defined as the first principal component of a given module and can, therefore, present the gene expression profiles in a module (Langfelder and Horvath, 2008).

### Association Between Modules and Clinical Information

Pearson's correlation analysis was applied to correlate the atrial rhythms with the MEs from each module. Associations between modules and AF susceptibility were determined by evaluating the correlations between the MEs and the atrial rhythm phenotypes in the 117 AF/SR and SR/SR samples (AF/SR was assigned one and SR/SR was assigned zero). Similarly, associations between modules and AF persistence were determined by evaluating the correlation between MEs and atrial rhythm phenotypes in the 192 AF/AF and AF/SR samples (AF/AF was assigned one and AF/SR was assigned zero). To reduce the probability of statistical error, we only chose the four most significant modules associated with AF (two for susceptibility and two for persistence) for further analysis.

### Preparation of miRNA Targets Database

DIANA-LncBase v2.0 (http://www.microrna.gr/LncBase) is a reference repository that contains experimentally supported non-coding RNA–miRNA pairs (Paraskevopoulou et al., 2016). DIANA-TarBase v8 (http://www.microrna.gr/tarbase) is a reference database with experimentally supported mRNA-miRNA pairs (Karagkouni et al., 2018). We annotated the Ensemble ID identifiers in the two data sets, as described above, and retained only the identified lncRNA–miRNA and mRNA–miRNA pairs, resulting in 26,178 lncRNA–miRNA pairs and 418,758 mRNA–miRNA pairs, which were used for further ceRNA pair prediction.

### ceRNA Network Construction

For each of the selected modules, the mRNAs and lncRNAs were co-expressed and closely related to AF. Thus, we predicted their communications by identifying shared miRNAs between lncRNAs and mRNAs. We used a strict prediction and selection method as follows: (a) prediction of lncRNA–miRNA interactions; (b) prediction of mRNA–miRNA interactions; (c) hypergeometric test: for each lncRNA–mRNA pair with shared miRNAs, we calculated the pair's significance by performing a hypergeometric test using the phyper function in the stata packages in R software. The *p*-values were calculated as follows:

$$\begin{array}{l} \text{p}=1-\sum_{k=0}^{t-1}\frac{C_{M}^{k}C_{N-M}^{n-k}}{C_{N}^{n}}, \end{array}$$

where *N* represents the total number of miRNAs in the prepared lncRNA–miRNA and mRNA–miRNA pairs (*N* = 923), *t* represents the number of shared miRNAs between the given lncRNA and mRNA, *n* represents the number of miRNAs that target the lncRNA, and *M* represents the number of miRNAs that target the mRNA. Those pairs with *p* < 0.05 were selected to construct the ceRNA network. We also calculated an adjusted *p*-value using the Benjamini–Hochberg (BH) method. Those pairs with adjusted *p* < 0.05 were used in the following sensitivity analysis.

### Enrichment Analysis

For a given gene list, a gene ontology biological process (GO BP) enrichment analysis was conducted using the ClusterProfiler (Yu et al., 2012) package in R software.

### Random Walk With Restart on Multiplex Networks

RWR-M is a state-of-the-art algorithm in network computational biology (Valdeolivas et al., 2019). It can be applied to prioritize candidate disease genes by calculating the proximity score of other nodes to known disease genes (seed nodes) in multiple interaction networks. Consider a multiplex graph *G* of L undirected graphs that share the same sets of n nodes. Each layer α = 1,…L, is defined by its n × n adjacency matrix ${\text{A}}^{\left[\alpha \right]}={\left({\text{A}}^{\left[\alpha \right]}(i,j)\right)}_{i,j=1,\ldots n}$, where A^[α]^(*i, j*) = 0 if nodes *i* and *j* are not directly connected on layer α and one otherwise. Specifically, A^[α]^(*i, i*) = 0, *i* = 1, …*n*. The multiplex graph is then defined as G = (V, E), where $\text{V}=\left\{v_{i}^{\alpha }i=1,\ldots n,\alpha =1,\ldots \text{L}\right\}$; $\text{E}=\left\{\left(v_{i}^{\alpha }v_{j}^{\alpha }\right),i,j=1,\ldots n,\alpha =1,\ldots {\text{L},\text{A}}^{\left[\alpha \right]}\left(i,j\right)\neq 0\right\}⋃\;\left\{\left(v_{i}^{\alpha },v_{i}^{\beta }\right),i=1,\ldots n,\alpha \neq \;\beta \right\}.$

For each iteration, an imaginary particle can walk from its current node $v_{i}^{\alpha }$ to its neighbors within a layer or jump to $v_{i}^{\beta }$ (α ≠ β) in another layer. An *nL* × *nL* matrix M can be defined as

$$\begin{array}{l} \text{M}=\left(\begin{array}{cccc} \left(1-\delta \right)A^{\left[1\right]} & \frac{\delta }{L-1}I\; & \ldots & \frac{\delta }{L-1}I\\ \frac{\delta }{L-1}I & \left(1-\delta \right)A^{\left[2\right]} & \ldots & \frac{\delta }{L-1}I\\ \vdots & \vdots & \ddots & \vdots \\ \frac{\delta }{L-1}I & \frac{\delta }{L-1}I & \ldots & \left(1-\delta \right)A^{\left[L\right]} \end{array}\;\right), \end{array}$$

where I is the n × n identity matrix. The parameter δ ∈ [0, 1] is the probability of staying in a layer or jumping to another layer and was set as 0.5 in this study. The RWR-M equation can be defined as

$$\begin{array}{l} {\bar{\text{p}}}_{t+1}^{T}=\left(1-r\right)M{\bar{\text{p}}}_{t}^{T}+r{\bar{\text{p}}}_{RS}^{T}, \end{array}$$

where *M* is the column normalization transition matrix of *M*; ${\bar{\text{p}}}_{t}^{T}=\left[{\text{p}}_{t}^{1},\ldots {\text{p}}_{t}^{L}\right]$ and ${\bar{\text{p}}}_{t+1\;}^{T}=\left[{\text{p}}_{t+1}^{1},\ldots {\text{p}}_{t+1}^{L}\right]$ are n × L vectors with each element representing the probability of the walking particle in G; r ∈ [0, 1] is the restart probability that the particle can restart by jumping to seed nodes at each iteration in the graph and was set as 0.7. The ${\bar{\text{p}}}_{RS}$ is defined as ${\bar{\text{p}}}_{\text{RS}}$ = τ $\cdot {\bar{\text{p}}}_{0}$, where ${\bar{\text{p}}}_{0}$ represents the initial probability distribution. The seed nodes are assigned 1/*k* (*k* is the number of seeds), and other nodes are assigned zero. τ = [τ1,., τL] is the measurement of each layer's weight. After enough iterations, the difference between ${\bar{\text{p}}}_{t+1}$ and ${\bar{\text{p}}}_{t}$ becomes negligible, and the stationary probability distribution is reached. The elements in the distribution matrix then represent a proximity measure from every node to the seed(s). Nodes with a high “global proximity score” were, therefore, prioritized as new disease genes.

First, the lncRNA–mRNA pairs in the four ceRNA networks were aggregated into a single large network, which was set as the first layer. We then used the top 50% of evidence-supported gene–gene interactions with the highest confidence scores from the cardiac muscle data identified by the GIANT project (Greene et al., 2015). The GIANT project collected genome-wide, functional interaction networks in tissue- and cell-specific manners for more than 100 human tissues and cell types. We only extracted those edges for which the two nodes both existed in the aggregated ceRNA network. Then, we conducted the RWR-M algorithm with two layers (L = 2, aggregated ceRNA network, and GIANT-guided network) using the RandomWalkRestartMH package (Valdeolivas et al., 2019). For the RWR-M analysis, seed nodes must first be defined. We searched the DISEASE (Pletscher-Frankild et al., 2015), DisgeNET (Piñero et al., 2017), and MALACARD (Rappaport et al., 2017) databases to identify known AF protein-coding genes, and those presented in the network were set as seed nodes. For RWR-M, the layer weight was set to $\tau =\left[\frac{2}{\left(1+R\right)},\;\frac{2\times R}{\left(1+R\right)}\right],\;$where *R* is the ratio of the number of ceRNA-guided interactions in the first layer to the number of GIANT project-guided interactions in the second layer. After performing the RWR-M algorithm, the top two scoring lncRNAs were selected as the key lncRNAs associated with AF.

### Gene Set Variation Analysis

To further investigate the function of the prioritized lncRNAs and eliminate the effects of atrial rhythm, we conducted a GSVA. GSVA (Hänzelmann et al., 2013) is an unsupervised method that computes the enrichment score of a given gene set in each sample. We downloaded the latest GO BP gene sets from the Molecular Signatures Database v7.2 (https://www.gsea-msigdb.org/) (Mootha et al., 2003) and excluded those with gene sizes smaller than 10 or larger than 500. For each gene set, we identified the correlation with the prioritized lncRNAs by fitting a linear model as the GSVA score–atrial rhythm + expression value of lncRNA. The regression coefficient and *p*-value for each lncRNA were calculated using the stata package.

### Construction of Random Forest Classifiers and Validation

For each prioritized lncRNA, the expression values of the lncRNA and its mRNA pairs were used to construct a random forest classifier using the randomForest package (Liaw and Wiener, 2002) in R. The performance of the classifier was first validated in the training set using a six-fold cross-validation method and was further evaluated using an independent test data set. First, because RNA sequencing and microarray results can be characterized by substantial heterogeneity, the expression values of selected features in 118 AF/AF samples, 43 SR/SR samples, and 38 microarray samples were merged, and batch effects were removed using the combat function of the sva package in R, without specifying the covariate of interest (AF or SR). The RNA sequencing samples were used as the training set, and the remaining 38 microarray samples were used as the test set. The performance of the established classifier was evaluated using the receiver operating characteristic curve and the value of the area under the curve (AUC).

## Results

### Data Preparation

After data preprocessing, we obtained a large gene expression matrix consisting of 16,905 unique genes (1,994 lncRNAs and 14,911 mRNAs) rows by 235 sample columns (AF/AF, *n* = 118; AF/SR, *n* = 74; and SR/SR, *n* = 43). An SV analysis was performed to identify potential large effectors of gene expression that might potentially introduce spurious correlations between genes, including technical variables, such as batch effects and read depths; genetic variables; and environmental variables, such as any history of structural heart disease and age (Leek and Storey, 2007; Leek et al., 2010; Parsana et al., 2019). Two SVs were identified when specifying the interest of atrial rhythm and covariate of sex. We then fit the gene expression matrix with a linear model that included atrial rhythm, sex, and the identified SVs (expression–atrial rhythm + sex + SVs) and regressed out the sex variable and the SVs. The cleaned expression matrix was used for further analyses, and the GSE41177 data set was used for validation.

### Construction of Co-Expression Modules and the Identification of Key Modules

**Figure 2:**
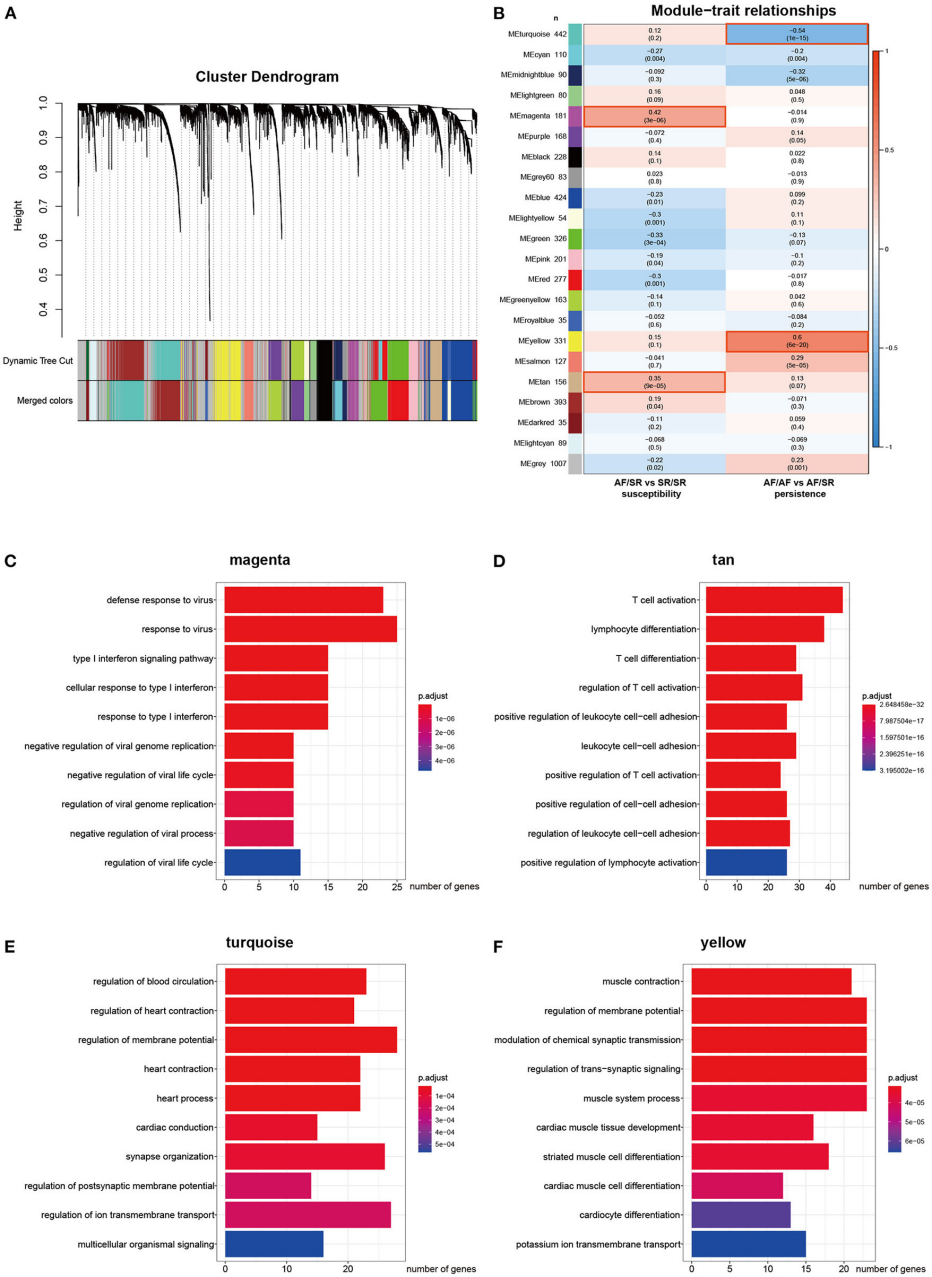
Construction of the weighted co-expression network and module analysis. **(A)** Cluster dendrogram. The two colored rows below represent the original and merged modules. **(B)** Heat map of the correlation between AF susceptibility/persistence and module eigengenes. Each row corresponds to a different module eigengene, and each column corresponds to a different AF trait. Each cell contains the corresponding correlation (first line) and *p*-value (second line). **(C–F)** Top 10 enriched biological processes associated with the AF-related modules.

The top 5,000 genes with the highest variation (including 460 lncRNAs and 4,540 mRNAs) were selected for the construction of a gene co-expression network. The β value was set to 12. A total of 21 modules were generated using dynamic tree cutting (Figure 2A). We then analyzed the correlation between each module and the clinical traits by calculating the correlation coefficient between each ME and the atrial rhythm phenotype. As shown in Figure 2B, the magenta (*r* = 0.42, *p* = 3e−6) and tan (*r* = 0.35, *p* = 9e−5) modules represent the top two AF susceptibility-associated modules, whereas the turquoise (*r* = −0.54, *p* = 1e−15) and yellow (*r* = 0.6, *p* = 6e−20) modules represent the top two AF persistence-associated modules. In addition, the magenta and tan modules did not significantly correlate with AF persistence, and the turquoise and yellow modules did not significantly correlate with AF susceptibility, indicating that the selected modules each have specificity for either AF susceptibility or persistence. Supplementary Table 1 summarizes the basic and functional information for all 21 modules. Figures 2C–F show the top 10 GO BP enrichment results for the four selected modules. The magenta module is primarily associated with the type I interferon signaling pathway, and the tan module is primarily associated with T cell activation. Both the turquoise and yellow modules are associated with muscle contraction and the regulation of membrane potential. These results indicate that immune system activation is closely associated with AF susceptibility, and electrical remodeling is more closely associated with AF persistence.

### Construction of ceRNA Networks in a Module-Specific Manner

**Figure 3:**
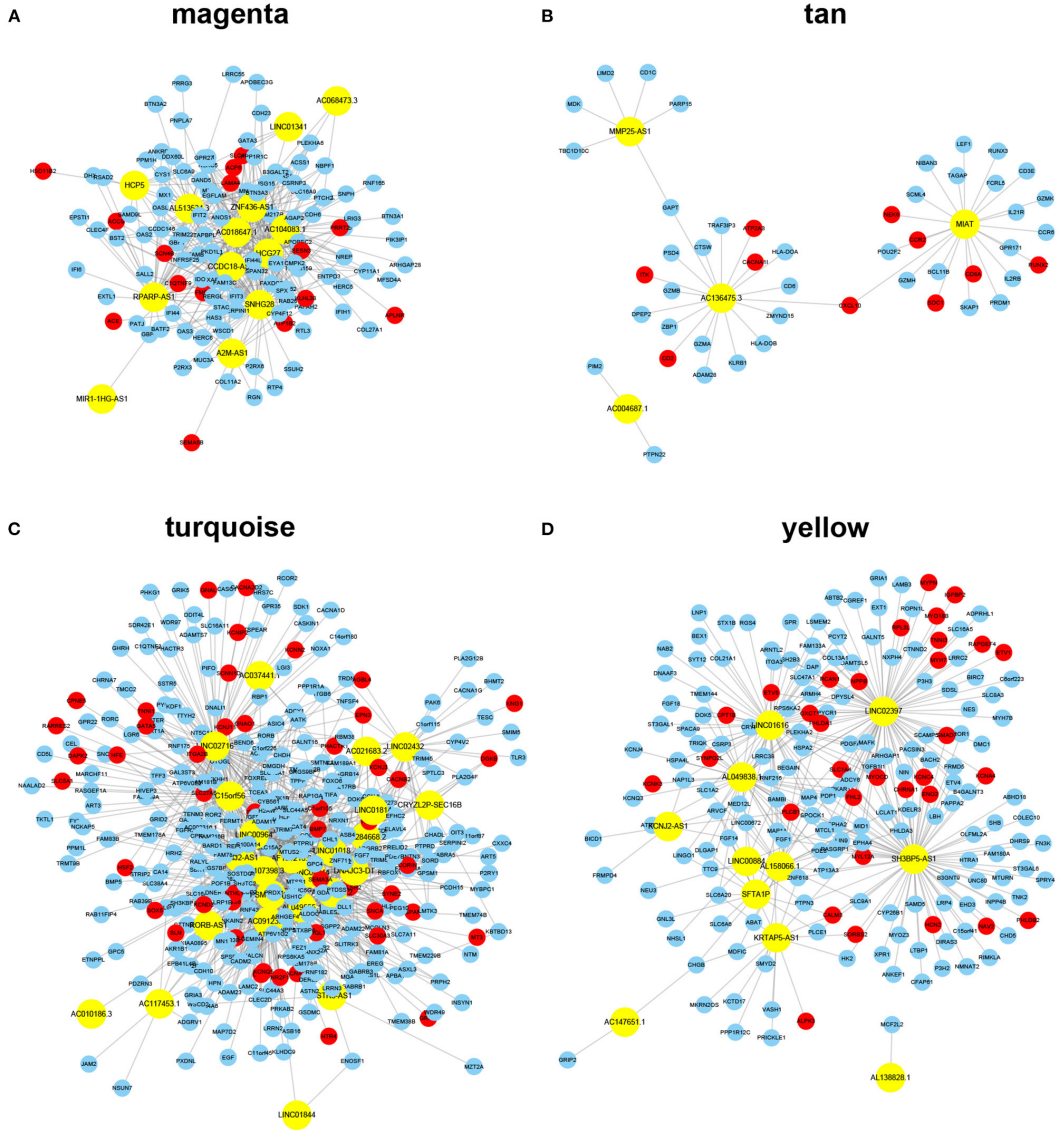
ceRNA networks for each module. Note: the yellow color represents lncRNAs, whereas the red color represents known AF protein-coding genes identified from DISEASE, DisgeNET, and MALACARD databases. **(A–D)** lncRNA-mRNA ceRNA networks identified using a module-specific manner in AF susceptibility-associated magenta **(A)** and tan **(B)** modules, and AF persistence-associated turquoise **(C)** and yellow **(D)** modules.

**Figure 4:**
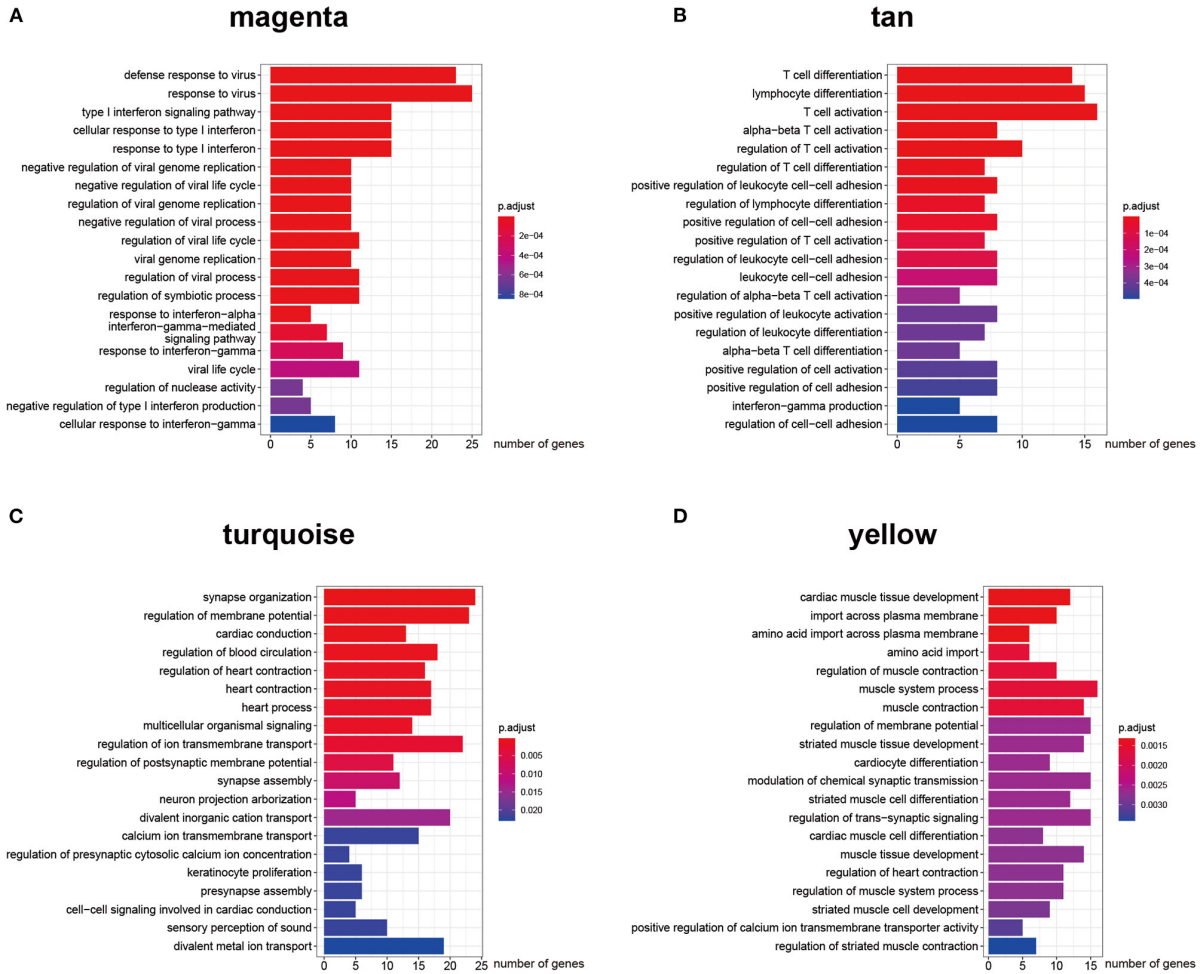
Gene ontology biological process enrichment analysis for each ceRNA network. **(A–D)** Top 20 enriched gene ontology biological process terms of protein-coding genes in AF susceptibility-associated magenta **(A)** and tan **(B)** ceRNA networks, and AF persistence-associated turquoise **(C)** and yellow **(D)** ceRNA networks.

The key design of our study was the construction of the ceRNA network among highly co-expressed genes. We reasoned that, if two genes exist in different modules, their ceRNA interactions would likely be less strong. Thus, we did not consider any intermodule ceRNA pairs. For each of the four AF modules, we identified the intramodule lncRNA–mRNA ceRNA pairs through the prediction and selection methods described in section Association between modules and clinical information. We obtained four independent ceRNA networks, two associated with AF susceptibility and two associated with AF persistence (Figure 3). The GO BP enrichment analysis (Figure 4) shows that the magenta ceRNA network was primarily associated with the defense response to virus and type I interferon signaling pathway, the tan ceRNA network was associated with T cell differentiation and T cell activation, the turquoise ceRNA network was primarily associated with synapse organization and regulation of membrane potential, and the yellow ceRNA network was primarily associated with cardiac muscle tissue development and muscle contraction.

We successfully constructed two inflammation-associated ceRNA networks related to AF susceptibility and two cardiac conduction or electrical remodeling-associated ceRNA networks related to AF persistence. Based on Figure 3, the majority of the nodes in each network appear to be able to communicate with each other through ceRNA language-guided interactions, either directly (lncRNA–mRNA) or indirectly (an mRNA–lncRNA–mRNA–lncRNA axis). These lncRNAs and mRNAs represent valuable therapeutic targets to prevent AF progression as they were not only co-expressed but also functionally correlated. Moreover, a total of 106 previously identified AF protein-coding genes were included in the established ceRNA networks (Figure 3, red nodes), including 49 in the turquoise ceRNA network, 32 in the yellow ceRNA network, 15 in the magenta ceRNA network, and 10 in the tan ceRNA network. The hypergeometric test showed a *p*-value of 8e−10 for the AF enrichment. These findings further demonstrate the significance of the constructed networks. More interestingly, 16 of the identified protein-coding genes have been implicated in the GWAS of AF conducted by Roselli et al. (2018, 2020) and Nielsen et al. (2018), including *AGBL4, COG5, DGKB, HSF2, KCND3, KCNN2, SLC27A6, SYNE2*, and *SYNPO2L* in the turquoise ceRNA network and *MYH7, MYOCD, MYO18B, NAV2, PHLDB2, RPL3L*, and *SMAD7* in the yellow ceRNA network. These results indicate that the roles of these GWAS-related genes in AF are likely associated with atrial electrical remodeling. Detailed information for each ceRNA network, including shared miRNAs between any lncRNA–mRNA pair, can be found in Supplementary Table 2.

### Prioritizing Key lncRNAs Associated With AF Using the RWR-M Algorithm

**Figure 5:**
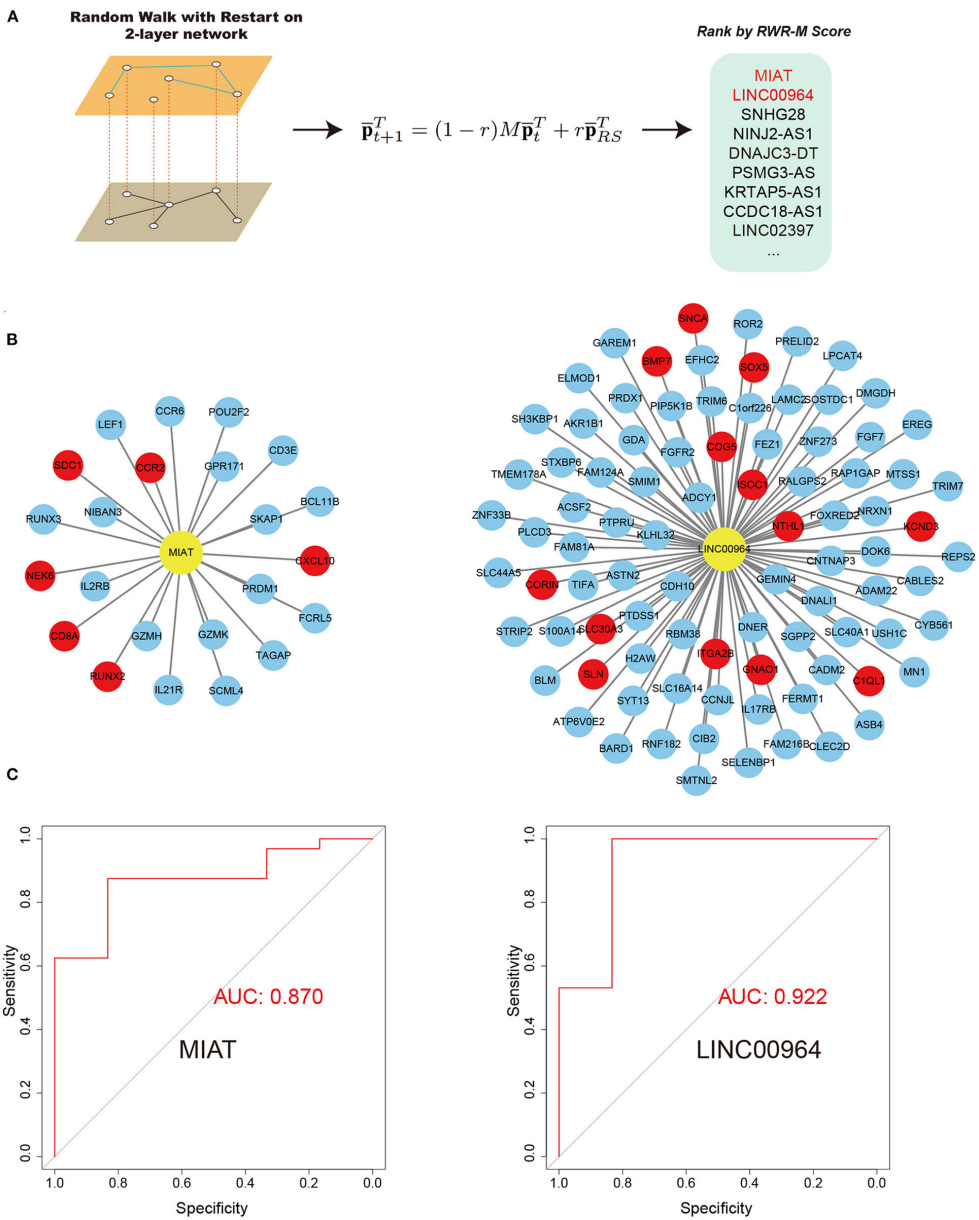
RWR-M algorithm and the performance of random forest classifiers in the test set. **(A)** Process of RWR-M. **(B)** MIAT-related ceRNA pairs (left) and LINC00964-related ceRNA pairs (right). Yellow color represents lncRNAs and red color represents known AF protein-coding genes. **(C)** Performance of the MIAT-related ceRNA pairs-based classifier (left) and the LINC00964-related ceRNA pairs-based classifier (right), as reflected by their respective receiver operating characteristic curves.

We then aimed to prioritize key lncRNAs associated with AF (Figure 5). We defined the hub lncRNAs with high graphical proximity to known AF genes. We applied the latest RWR-M algorithm with two different layers (the aggregated ceRNA network and the GIANT project-guided network; Figure 5A). The calculated RWR-M score can be considered a measure of the proximity between the seed(s) and all other nodes in the graph. Those genes with high RWR-M scores are then identified as hub genes. After performing the RWR-M algorithms, MIAT and LINC00964 were identified as the genes with the top two highest RWR-M scores and were identified as hub genes associated with AF (Figure 5A; scores for all lncRNAs in the network and their diagnostic efficiency for distinguishing AF from SR are described in Supplementary Table 3). We also tested whether using BH-adjusted *p*-values in step 2.5 would substantially change the results. After filtering lncRNA–mRNA pairs with an adjusted *p* < 0.05 and conducting the subsequent protocols, MIAT and LINC00964 remained among the top five high RWR-M scoring lncRNAs. The lncRNA MIAT belongs to the AF susceptibility-associated tan module, whereas the lncRNA LNIC00964 belongs to the AF persistence-associated turquoise module. The miRNA partners of MIAT-mediated ceRNA pairs include 19 miRNAs (Supplementary Table 2), some of which have previously been implicated in AF, such as miR-27b-3 (Lv et al., 2019) and miR-23b-3p (Yang et al., 2019). The miRNA partners of LINC00964-mediated ceRNA pairs only included miR-34a-5p. Interestingly, a previous study has demonstrated that miR-34a promoted atrial electrical remodeling by enhancing intracellular Ca^2+^ signaling (Zhu et al., 2018). These results revealed that these miRNA-mediated ceRNA pairs likely served critical roles during AF development.

### Identifying Biological Processes Correlated With MIAT and LINC00964

The lncRNA MIAT has 23 direct mRNA targets in the tan module, including six known AF genes. GO BP enrichment analysis identified the 23 genes associated with MIAT as being primarily involved in lymphocyte differentiation and T cell activation (data not shown). The lncRNA LINC00964 has 89 direct mRNA targets in the turquoise module, including 13 known AF genes. No significant term was enriched among these 89 mRNAs based on Fisher's exact test with BH adjustment, indicating that the 89 direct targets of LINC00964 have multiple biological functions. These genes are significantly associated with atrial electrical remodeling because they (1) were co-expressed with atrial electrical remodeling-related genes in the turquoise ceRNA network and (2) co-interacted with atrial electrical remodeling-related genes in the turquoise ceRNA network through ceRNA-guided interactions. To further investigate the biological functions of MIAT and LINC00964, we conducted a GSVA and correlated the GSVA score of each pathway (a total of 5,348 gene sets) with the expression value of each lncRNA while adjusting for the AF phenotype (Supplementary Table 4 for MIAT and Supplementary Table 5 for LINC00964). For MIAT, all of the top 10 positively correlated pathways were identified as T cell activation–related pathways, with T_Helper_17_Cell_Lineage_Commitment having the highest regression coefficient of 0.384. For LINC00964, the top 10 positively correlated gene sets were primarily involved in rhythm control-related processes. In addition, nearly all of the cardiac conduction–associated pathways were significantly positively correlated with LINC00964. For example, the Atrial_Cardiac_Muscle_Cell_Membrane_Repolarization pathway was correlated with LINC00964 with a regression coefficient of 0.178 and a *p*-value of 0.015. Taken together, these findings suggest that MIAT was closely associated with T cell activation, whereas LINC00964 was closely associated with atrial electrical remodeling.

### Establishing a Classifier Based on Key lncRNA–mRNA Pairs

We reasoned that if MIAT and LINC00964 were important in AF, the expression values of their related ceRNA pairs would have the ability to discriminate persistent AF from SR. For each of the two lncRNAs, we extracted related ceRNA pairs in the network (Figure 5B) and constructed a classifier based on their expression values, using random forest algorithms. Before the random forest algorithm, we removed the batch effects associated with the microarray samples and the RNA sequencing samples that did not fit the interest (AF or SR) into the model to increase the power of the classifier as the microarray data and RNA sequencing data have high heterogeneity. The classifiers were first validated in the training set using a six-fold cross-validation method. Both MIAT-based and LINC00964-based classifiers showed a strong ability to discriminate AF from SR in the training set with AUC values of 0.810 and 0.940, respectively. We then evaluated their performances in the independent microarray data set. Promisingly, high AUC values were obtained for both classifiers, including 0.870 for the MIAT-based classifier and 0.922 for the LINC00964-based classifier (Figure 5C). These results further indicate the importance of these ceRNA pairs in AF pathogenesis and might provide new diagnostic tools for AF. In the sensitivity analysis, we determined the effects of retaining the batch effects, which resulted in the AUC values of the MIAT- and LINC00964-based classifiers for the independent test set being reduced to 0.75 and 0.70, respectively. This result highlights the importance of removing batch effects before constructing classifiers.

## Discussion

To our knowledge, this is the first study to identify AF susceptibility- and persistence-specific gene modules and ceRNA networks. The large sample size ensures that these results are more reliable than most previously conducted AF bioinformatic studies. By comparing the MEs from patients in SR who differed according to a history of previous AF (AF/SR vs. SR/SR), we identified two co-expression modules associated with AF susceptibility, both of which were primarily associated with inflammatory processes. By comparing the MEs from patients in AF rhythm with those from patients with a history of AF but in SR (AF/AF vs. AF/SR), we identified two co-expression modules associated with AF persistence, both of which primarily associated with the processes of electrical remodeling. These results were consistent with those of a previous study that compared the genome-wide mRNA microarray profiling of LAA tissues between AF/AF, AF/SR, and SR/SR patients (Deshmukh et al., 2015), in which altered transcriptional activity associated with inflammation and the remodeling of ion channel expression were also associated with AF susceptibility and persistence, respectively.

Our next goal was to construct ceRNA networks based on gene co-expression modules, which differed from a previous study (Qian et al., 2019) that used identified DEGs to predict AF-associated ceRNA pairs, which does not account for any interactions between genes. For each ceRNA network, the nodes were not only co-expressed, but also interacted functionally, and most of the nodes could communicate either directly or indirectly. The function of each network was closely associated with a specific feature of atrial remodeling, which could provide a better understanding of gene functions. For example, the identification of a gene in the turquoise ceRNA network could indicate a role in AF through the regulation of genes involved in electrical remodeling. This information might also help to relate genetics with epigenetics and disease phenotypes as 16 AF-GWAS-related genes were identified in the currently constructed electrical remodeling-related networks.

Another innovation of the present study was the application of the state-of-the-art RWR-M algorithm, which is an improvement on the RWR algorithm, to prioritize lncRNAs. Most previous studies (Song et al., 2016; Qian et al., 2019; Wang et al., 2020) use the RWR algorithm to identify new disease genes based on a single network. However, this approach ignores functional interactions, such as co-expression networks and co-annotation networks, and each type of network has different relationships, advantages, and biases (Lee et al., 2019). One should note that the RWR-M algorithm differs from the simple aggregation of various types of interactions into an aggregated network, which would dismiss the individual features and topologies of each network and has been shown to be less effective for prioritizing new disease genes than the RWR-M algorithm (Valdeolivas et al., 2019). By applying the RWR-M algorithm based on ceRNA language-guided and evidence-supported interactions identified by a previously published data set (Greene et al., 2015), MIAT and LINC00964 were identified as the top two genes with the highest proximities to known AF genes. After adjusting for the covariate of atrial rhythm, MIAT was significantly correlated with T cell activation, especially T helper 17 cells, whereas LINC00964 was correlated with rhythm control. Moreover, LINC00964 was also significantly correlated with the atrial electrical remodeling–related process. Using the random forest algorithm, we further demonstrated that their associated ceRNA pairs could distinguish persistent AF from SR patients in both the training set and an independent test set, further indicating the importance of MIAT and LINC00964 in AF susceptibility and persistence, respectively.

MIAT has been shown to promote cardiac fibrosis through the MIAT/miR-24/Furin/transforming growth factor (TGF)-beta 1 axis (Qu et al., 2017), promote cardiac hypertrophy through the miR-150/P300 axis (Zhu et al., 2016) and miR-93/TLR4 axis (Li et al., 2018), promote extracellular matrix deposition through the miR-29/COL1A1 and COL3A1 axes (Chuang et al., 2020), and regulate vascular endothelial cell function through the miR-150-5p/VEGF axis (Yan et al., 2015). Our study indicates that MIAT is a T cell activation–associated lncRNA, especially Th17 cells, and is closely associated with AF susceptibility. This corroborates a recent study that found that cinnamaldehyde can ameliorate ulcerative colitis through the suppression of Th17 cells and the regulation of MIAT (Qu et al., 2020). The knockdown of MIAT has been shown to alleviate the inflammatory response and reduce intracellular oxidative stress in LPS-stimulated atrial HL-1 cells (Xing et al., 2020) and attenuate AF and AF-induced atrial fibrosis by targeting miR-133a-3p (Yao et al., 2020). Th17 cells are also suggested to contribute to AF susceptibility. Elevated plasma levels of Th17-related cytokines have been associated with a high risk of AF (Wu et al., 2016), and serum IL-17A levels are associated with AF recurrence (Xu et al., 2019). Experiments have also shown that Th17-produced IL-17A contributes to the development of AF by promoting inflammation and cardiac fibrosis (Fu et al., 2015). Other types of T cells, such as Th1, Th2, and Tregs, have also been implicated in AF (Liu et al., 2018). These studies are consistent with our finding and suggest the involvement of MIAT in AF through the mediation of T cell activation and inflammation. Targeting MIAT might prevent AF occurrence and recurrence.

We also identified LINC00964 as a central AF persistence-related lncRNA that is closely associated with atrial electrical remodeling. The function of LINC00964 has not previously been investigated. However, by searching the results of GWAS, we found that an AF-related single-nucleotide polymorphism (SNP), rs35006907, exists in the intronic region of LINC00964. The presence of SNPs in the promoter, intronic, or exonic regions of lncRNAs can affect transcription, structure, or function (Castellanos-Rubio and Ghosh, 2019). For example, the SNP rs11672691, located in the promoter region of lncRNA PCAT19, modulates the function and expression of PCAT19, promoting prostate cancer progression through the upregulation of cell cycle gene expression (Hua et al., 2018). By searching the expression quantitative trait loci (eQTLs) results from the Genotype-Tissue Expression (GTEx) consortium database (http://www.gtexportal.org/home/) version 8 (GTEx Consortium, 2015), rs35006907 was identified as being negatively correlated with LINC00964 expression levels, in both heart-atrial appendage tissue (normalized effect size = −0.28, *p* = 2.7e−8) and heart-left ventricle tissue (normalized effect size = −0.35, *p* = 4.3e−13). This direct evidence between SNP and gene expression indicates that the r35006907 SNP could promote AF persistence by negatively regulating the expression of LINC00964. The downregulation of LINC00964 expression would promote AF electrical remodeling by affecting the expression of ion channel–related genes in the turquoise ceRNA network.

Several limitations should be acknowledged in this study. First, the RNA sequencing technique that was used for GSE68868 was not specialized for the identification of lncRNAs; thus, only a small number of lncRNAs were available after filtering out low-expression genes. Second, lncRNAs can affect protein-coding gene function through diverse pathways, and we only considered the effects of the ceRNA mechanism. Third, lncRNAs or mRNAs can also communicate with each other through the ceRNA language, which was not analyzed. Finally, no attempt was made to validate the functions of the identified ceRNA pairs using an experimental model, and the causal relationships remain unclear.

In conclusion, our study constructed AF susceptibility- and persistence-associated ceRNA networks, identified relationships between genetic and epigenetic pathways, prioritized MIAT and LINC00964 as key lncRNAs, and constructed random forest classifiers based on their associated ceRNA pairs. These results will help us to better understand the mechanisms underlying AF from the ceRNA perspective and provide candidate therapeutic and diagnostic tools.

## Data Availability Statement

The original contributions presented in the study are included in the article/Supplementary Material, further inquiries can be directed to the corresponding author/s. A copy of the code used in this analysis is available at: https://github.com/Yaozhong-Liu/ceRNA_in_af.

## Author Contributions

YL performed the bioinformatic analysis and was a major contributor in writing the manuscript. NL and FB made important modifications to the manuscript. YL and QL designed the research project and created the final revision of the manuscript. All authors read and approved the final version of the manuscript.

## Conflict of Interest

The authors declare that the research was conducted in the absence of any commercial or financial relationships that could be construed as a potential conflict of interest.
